# Clinical Evaluation of the Marginal Adaptation of Metal-Ceramic and All-Ceramic Fixed Partial Dentures Using Different Luting Cements

**DOI:** 10.7759/cureus.101777

**Published:** 2026-01-18

**Authors:** Jay Gohil, Minhaj Ahmed Amer Mohammed, Narendra Basutkar, Amith Madiramalingaiah Setty, Nilofer Halim, Rahul VC Tiwari, Heena Tiwari, Girija Dodamani, Seema Gupta

**Affiliations:** 1 Department of Prosthodontics, Crown and Bridge, K.M. Shah Dental College and Hospital, Sumandeep Vidyapeeth (Deemed to be University), Vadodara, IND; 2 Department of Oral and Maxillofacial Surgery, Mazaya Dental Center, Muhayil, SAU; 3 Department of Prosthodontics, Ibn Sina National College for Medical Studies, Jeddah, SAU; 4 Department of Conservative Dentistry and Endodontics, SJM Dental College and Hospital, Chitradurga, IND; 5 Department of Oral Medicine and Radiology, Al-Azhar Dental College, Thodupuzha, IND; 6 Department of Oral and Maxillofacial Surgery, RKDF Dental College and Research Center, Bhopal, IND; 7 Department of Blood Cell, Commissionerate of Health and Family Welfare, Hyderabad, IND; 8 Department of Prosthodontics, Crown and Bridge, Jawahar Medical Foundation's Annasaheb Chudaman Patil Memorial (ACPM) Dental College and Hospital, Dhule, IND; 9 Department of Orthodontics, Kothiwal Dental College and Research Centre, Moradabad, IND

**Keywords:** adaptation, cement, ceramics, fixed partial denture, marginal

## Abstract

Introduction

Marginal adaptation is a key factor in the biological and mechanical success of fixed partial dentures (FPDs). This study aimed to clinically compare the marginal adaptation of metal-ceramic and all-ceramic FPDs cemented with resin-modified glass ionomer cement (RMGIC) and self-adhesive resin cement.

Materials and methods

This prospective clinical observational study included 60 patients who required three-unit posterior FPDs. The patients were allocated into two groups: metal-ceramic (n = 30) and all-ceramic (n = 30). Each group was subdivided according to the luting cement used: RMGIC or self-adhesive resin cement (n = 15 each). Standardized clinical and laboratory protocols were used. Marginal adaptation was assessed using the modified United States Public Health Service (USPHS) criteria immediately after cementation and at three months by calibrated, blinded examiners. Data were analyzed using non-parametric tests and analysis of covariance (ANCOVA), with significance set at p < 0.05.

Results

At baseline, all groups demonstrated a clinically ideal marginal adaptation, with a median USPHS score of 1. Mean baseline scores ranged from 0.73 to 1.00 across subgroups. At three months, metal-ceramic FPDs cemented with RMGIC showed the highest mean USPHS score (2.6 ± 0.51), indicating greater marginal discrepancy. All-ceramic FPDs cemented with self-adhesive resin cement demonstrated the lowest mean score (0.87 ± 0.83), followed by metal-ceramic FPDs with self-adhesive resin cement (1.73 ± 0.70). Intergroup comparisons showed significantly better marginal adaptation in all-ceramic restorations than in metal-ceramic restorations (p < 0.001). Cement type also significantly influenced the outcomes, with self-adhesive resin cement performing better than RMGIC (p < 0.001). No significant interaction was observed between the restoration type and cement type (p = 0.913).

Conclusion

All-ceramic FPDs demonstrated superior early marginal adaptation compared with metal-ceramic restorations. The use of self-adhesive resin cement resulted in improved marginal integrity irrespective of the restoration type.

## Introduction

Fixed partial dentures (FPDs) are one of the most widely used restorative treatment options for replacing missing teeth, offering favorable esthetics, function, and long-term predictability. The success and longevity of FPDs are influenced by multiple factors, among which marginal adaptation plays a critical role [1]. An optimal marginal fit is essential to prevent plaque accumulation, microleakage, secondary caries, periodontal inflammation, and eventual prosthesis failure. Even minor discrepancies at the tooth-restoration interface can compromise biological and mechanical outcomes, making marginal adaptation a key determinant of clinical success [2].

Metal-ceramic restorations have long been regarded as the gold standard for FPDs because of their excellent strength, durability, and long-term survival [3]. However, their esthetic limitations, including potential grayish margins and opacity, have led to the increasing adoption of all-ceramic systems [4]. Advances in ceramic materials, such as zirconia and lithium disilicate, have significantly improved the strength, translucency, and clinical performance of all-ceramic restorations, making them a preferred choice in contemporary prosthodontics [5].

Despite these advancements, concerns regarding the marginal integrity of different restorative systems continue to exist. While numerous in vitro studies have compared marginal gaps using laboratory-based measurement techniques, the clinical evaluation of marginal adaptation remains more relevant, as it reflects the true performance of restorations in the oral environment. However, limited clinical studies have directly compared the marginal adaptation of metal-ceramic and all-ceramic FPDs using standardized clinical assessment methods [6,7].

Therefore, the present study aimed to clinically evaluate and compare the marginal adaptation of metal-ceramic and all-ceramic FPDs by using accepted clinical assessment methods. The objectives of this study were to assess the marginal fit of both types of FPDs with two different luting cements, compare the frequency of marginal discrepancies between the two materials, and determine whether a statistically significant difference exists in their clinical marginal adaptation.

## Materials and methods

Study design and setting

This clinical comparative study was designed as a prospective observational study and was conducted at the Department of Prosthodontics, Jawahar Medical Foundation's Annasaheb Chudaman Patil Memorial Dental College and Hospital, Dhule, India. All clinical and laboratory procedures were performed under standardized conditions within institutional facilities. The study was conducted over a period of 12 months and included patient recruitment, clinical procedures, prosthesis fabrication, cementation, and marginal adaptation assessment. Prior to commencement, ethical approval was obtained from the Institutional Ethics Committee (EC/NEW/INST/2022/2959/SS102). This study was conducted in accordance with the Declaration of Helsinki for research involving human participants. All eligible participants were informed in detail about the nature, purpose, potential benefits, and risks of the study in their local languages. Written informed consent was obtained from each participant before inclusion in the study.

Eligibility criteria

Inclusion Criteria

Patients aged between 18 and 60 years who required a three-unit FPD for replacement of a single missing tooth in the posterior region, had good periodontal health, vital abutment teeth with adequate clinical crown height, and were willing to participate and provide informed consent.

Exclusion Criteria

Patients with parafunctional habits such as bruxism, poor oral hygiene, or active periodontal disease, medically compromised patients, teeth with inadequate periodontal support, long-span FPDs, and a history of allergy to dental restorative materials were excluded.

Sample selection and grouping

Power analysis was conducted using the G*Power software (version 3.1.9.2, Heinrich Heine University, Düsseldorf, Germany). To detect a clinically meaningful difference (medium effect size, d = 0.5) in marginal fit between metal-ceramic and all-ceramic crown groups, with 80% power and a 5% alpha level, a minimum total sample size of 60 was required and allocated equally into two groups of 30 each [8]. Sixty patients who fulfilled the inclusion criteria were selected using convenience sampling. The patients were randomly allocated using computer-generated random numbers into two groups: Group 1 received metal-ceramic FPDs (n = 30), and Group 2 received all-ceramic FPDs (n = 30).

Each primary group was further subdivided into two subgroups (n = 15 each) based on the type of luting cement used: Subgroup A, resin-modified glass ionomer cement (RMGIC); and Subgroup B, self-adhesive resin cement.

Tooth preparation and impression procedure

Standardized tooth preparation was performed on all abutment teeth under rubber dam isolation. For metal-ceramic FPDs, tooth preparation included a 1.5-2.0 mm occlusal reduction, 1.2 mm axial reduction, and a circumferential heavy chamfer finish line. For all-ceramic FPDs, a 2.0 mm occlusal reduction, 1.5 mm axial reduction, and a deep chamfer finish line were prepared. All the internal line angles were rounded.

Gingival retraction was performed using retraction cords (Ultrapak®, Ultradent Products Inc., South Jordan, Utah, USA) impregnated with a hemostatic agent (ViscoStat®, Ultradent Products Inc.). Final impressions were made by adding a silicone impression material (Aquasil®, Dentsply Sirona, York, Pennsylvania, USA) using a dual-mix single-stage technique. Type IV dental stone (Kalrock®, Kalabhai Karson Pvt. Ltd., Mumbai, India) was used for the master cast fabrication.

Fabrication of FPDs

The metal framework for the metal-ceramic FPDs was fabricated using a nickel-chromium alloy (Wiron 99®, BEGO GmbH, Bremen, Germany) using the lost-wax casting technique. Porcelain veneering was performed using feldspathic ceramic (Vita VMK Master®, VITA Zahnfabrik, Bad Säckingen, Germany).

For all-ceramic FPDs, zirconia frameworks were fabricated using computer-aided design/computer-aided manufacturing (CAD/CAM) technology from pre-sintered zirconia blanks (Cercon®, Dentsply Sirona, Bensheim, Germany). Veneering porcelain was applied using a compatible ceramic layering material (Cercon Ceram Kiss®, Dentsply Sirona). All prostheses were evaluated on the master cast and intraorally for proximal contact, occlusion, esthetics, and marginal fit before the final cementation.

Cementation procedure and clinical evaluation of marginal adaptation

Cementation of the FPDs was performed according to the subgroup allocation. In Subgroup A, restorations were cemented using RMGIC (GC FujiCEM 2®, GC Corporation, Tokyo, Japan). In Subgroup B, cementation was carried out using self-adhesive resin cement (RelyX™ U200, 3M ESPE, St. Paul, Minnesota, USA).

All cementations were performed under standardized conditions using finger pressure, followed by the removal of excess cement after the initial setting. The occlusion was reevaluated and adjusted as required. To minimize operator-related variability, all cementation procedures were performed by a single, experienced clinician.

Marginal adaptation discrepancies were evaluated clinically using a sharp explorer (No. 23 Explorer, Hu-Friedy, Chicago, Illinois, USA) and direct visual examination under dental operating light. The examiners were blinded to the crown type and the luting cement used. According to the modified United States Public Health Service (USPHS) criteria, restorations were graded as Alpha, Bravo, Charlie, or Delta based on their clinical condition. An Alpha grade represented a clinically ideal restoration with excellent marginal adaptation, no detectable gap on exploratory examination, absence of marginal discoloration, and no evidence of secondary caries, requiring no clinical intervention. A Bravo grade indicated a clinically acceptable restoration with a slight marginal discrepancy detectable by an explorer, but without dentin exposure, marginal staining, or recurrent caries, and therefore did not require immediate treatment. A Charlie grade was assigned to restorations that were clinically unacceptable due to a definite marginal gap, with exposed dentin, marginal breakdown, or evidence of early secondary caries, necessitating repair or corrective intervention. A Delta grade denotes the worst clinical condition, characterized by gross marginal failure, extensive secondary caries, loss of retention, fracture of the restoration, or other major defects, for which complete replacement of the restoration is required [9]. For USPHS criteria, licensed permission has been obtained for use in research and publication. Evaluation was performed immediately after cementation and at a follow-up of three months. Examiners assessing marginal adaptation were blinded to the type of restoration.

Calibration and reliability

Two independent examiners were calibrated prior to the study by evaluating 10 pilot cases that were not included in the main study. Inter-examiner and intra-examiner reliability were assessed using Cohen’s kappa statistic, and a kappa value greater than 0.80 was considered acceptable, indicating high agreement.

Outcome measures

The primary outcome measure was the clinical marginal discrepancies of metal-ceramic and all-ceramic FPDs cemented using different luting agents, assessed using the USPHS criteria [9]. The secondary outcome was the score of marginal adaptation, by Felton et al. [10], between the two groups at different abutment sites. Licensed permission has been obtained to use the scoring system for our research. Outcomes were assessed at baseline (T0) and after a three-month period (T1).

Statistical analysis

The USPHS discrepancies and marginal adaptation scores of both groups are presented as frequency, percentage, mean, median, and standard deviation. Data were analyzed using IBM SPSS Statistics for Windows, Version 25.0 (Released 2017; IBM Corp., Armonk, New York, USA). All data were analyzed for normal distribution using the Shapiro-Wilk test and confirmed as non-normal, which suggests the use of non-parametric tests (Mann-Whitney U test) for between-group comparisons. The Wilcoxon rank test was used for intragroup comparisons at multiple time points. An analysis of covariance (ANCOVA) test was applied for the overall comparison of the marginal adaptation of groups across time periods. Crown type and luting cement were considered fixed factors, whereas the baseline USPHS score was included as a covariate in the ANCOVA model. Statistical significance was set at p < 0.05. The effect size was also reported to indicate the strength of the analysis.

## Results

The demographic characteristics of the study groups are presented in Table 1. The overall metal-ceramic (n = 30) and all-ceramic (n = 30) groups were well-matched, with comparable mean ages (45.2 ± 8.1 vs. 44.7 ± 7.9 years) and sex distributions. Furthermore, within each crown type, the participants allocated to the two cement subgroups (RMGIC and self-adhesive cement, n = 15 each) were demographically similar. Minor variations in mean age between the cement subgroups were found, and the sex distribution was identical within each crown type.

**Table 1:** Demographic characteristics of participants according to crown type and luting cement. Values are presented as mean ± standard deviation for age and number (percentage) for sex; percentages are calculated within each subgroup. No statistically significant differences were observed between groups at baseline (p > 0.05). GIC: glass ionomer cement

Variable	Metal-ceramic			All-ceramic		
	Resin-modified GIC (n = 15)	Self-adhesive (n = 15)	Overall (n = 30)	Resin-modified GIC (n = 15)	Self-adhesive (n = 15)	Overall (n = 30)
Age (years)	44.2 ± 5.1	46.2 ± 7.1	45.2 ± 8.1	42.2 ± 8.2	45.2 ± 6.8	44.7 ± 7.9
Males (%)	9 (30%)	9 (30%)	18 (60%)	8 (26.6%)	8 (26.6%)	16 (53%)
Females (%)	6 (20%)	6 (20%)	12 (40%)	7 (23.4%)	7 (23.4%)	14 (47%)

At baseline, no statistically significant difference in marginal discrepancies (USPHS grading) was found between RMGIC and self-adhesive cement for either all-ceramic or metal-ceramic crowns. Descriptively, for all-ceramic crowns, RMGIC showed a higher proportion of ideal Alpha margins, while self-adhesive cement had more Charlie and Delta ratings. In contrast, metal-ceramic crowns demonstrated an identical outcome distribution for both cements, consisting solely of Bravo and Delta scores. These baseline results indicate that, while cement choice did not create a significant difference initially, the crown material itself influenced the initial adaptation profile more distinctly (Table 2).

**Table 2:** Comparison of both the groups for marginal adaptation using USPHS grading at baseline. p > 0.05 denotes no statistical significance using the chi-square test. Scores are presented as frequency (n) and percentage (%). Source: USPHS grading [9]. USPHS: United States Public Health Service criteria; GIC: glass ionomer cement

Group	Baseline grade	Subgroup		Intragroup comparison		Intergroup comparison	
		Resin-modified GIC, n (%)	Self-adhesive cement, n (%)	Chi-square statistic	p-value	Chi-square statistic	p-value
All-ceramic	Alpha	7 (23.33%)	3 (10.00%)	4.1	0.251	3.78	0.286
	Bravo	5 (16.66%)	5 (16.66%)				
	Charlie	0 (0.00%)	2 (6.66%)				
	Delta	3 (10.00%)	5 (16.66%)				
Metal-ceramic	Alpha	0 (0.00%)	0 (0.00%)	0	1		
	Bravo	8 (26.66%)	8 (26.66%)				
	Charlie	0 (0.00%)	0 (0.00%)				
	Delta	7 (23.33%)	7 (23.33%)				

At the three-month evaluation, cement choice significantly impacted marginal discrepancies (USPHS grading). For all-ceramic crowns, self-adhesive cement yielded superior outcomes (p = 0.01), with more ideal Alpha scores and no Delta scores, unlike RMGIC. The disparity was greater for metal-ceramic crowns. Self-adhesive cement produced acceptable Bravo margins and minimal Delta failures, while RMGIC resulted in no Alpha/Bravo scores and a high Delta failure rate. Overall, self-adhesive cement demonstrated significantly better, medium-term clinical performance for both crown types (Table 3).

**Table 3:** Comparison of both the groups for marginal adaptation using USPHS grading at three months. *p < 0.05 denotes statistical significance using the chi-square test. Scores are presented as frequency (n) and percentage (%). Source: USPHS grading [9]. USPHS: United States Public Health Service criteria; GIC: glass ionomer cement

Group	USPHS grade at three months	Subgroups		Intragroup comparison		Intergroup comparison	
		Resin-modified GIC, n (%)	Self-adhesive cement, n (%)	Chi-square statistic	p-value	Chi-square statistic	p-value
All-ceramic	Alpha	1 (3.33%)	6 (20.00%)	6.062	0.109	11.31	0.01*
	Bravo	6 (20.00%)	5 (16.66%)				
	Charlie	6 (20.00%)	4 (13.33%)				
	Delta	2 (6.66%)	0 (0.00%)				
Metal-ceramic	Alpha	0 (0.00%)	0 (0.00%)	10.53	0.005*		
	Bravo	0 (0.00%)	6 (20.00%)				
	Charlie	6 (20.00%)	7 (23.33%)				
	Delta	9 (30.00%)	2 (6.66%)				

The descriptive analysis revealed distinct patterns in marginal adaptation scores across crown and cement types over three months. At baseline, all groups started with a median score of 1; however, the all-ceramic/RMGIC subgroup had a notably lower mean score (0.73). At three months, all groups showed elevated scores (indicating potential degradation), but to varying degrees. Metal-ceramic crowns with RMGIC displayed the highest mean scores (2.6 ± 0.51), suggesting the greatest clinical change. In contrast, all-ceramic crowns with self-adhesive cement maintained the lowest mean score (0.87 ± 0.83), closely followed by their metal-ceramic counterpart (1.73 ± 0.7). The inference is that the choice of luting agent significantly influences medium-term clinical performance. Self-adhesive cement appears to be associated with better marginal adaptation and surface integrity (lower marginal adaptation scores) at three months for both crown types compared with RMGIC. This suggests a superior protective effect of resin cement, with all-ceramic crowns particularly benefiting from this combination (Table 4).

**Table 4:** Descriptive statistics of marginal adaptation scores at baseline and three-month follow-up according to crown and cement type. Lower scores indicate better marginal adaptation; scores were treated as continuous variables for descriptive purposes. Source: Scoring system for marginal adaptation was taken from a previous study by Felton et al. [10]. CI: confidence interval; N = 15 per subgroup; GIC: glass ionomer cement

Marginal adaptation scores	Group (Crown type)	Subgroup (Cement type)	N	Median	95% CI for mean	Mean ± SD
At baseline	Metal-ceramic	Resin-modified GIC	15	1	1.18 - 1.75	1.47 ± 0.52
		Self-adhesive cement	15	1	1.18 - 1.75	1.47 ± 0.52
	All-ceramic	Resin-modified GIC	15	1	0.29 - 1.18	0.73 ± 0.8
		Self-adhesive cement	15	1	0.85 - 1.95	1.4 ± 0.99
At three months	Metal-ceramic	Resin-modified GIC	15	3	2.32 - 2.88	2.6 ± 0.51
		Self-adhesive cement	15	2	1.34 - 2.12	1.73 ± 0.7
	All-ceramic	Resin-modified GIC	15	2	1.14 - 2.06	1.6 ± 0.83
		Self-adhesive cement	15	1	0.4 - 1.33	0.87 ± 0.83

The results of the two-way ANCOVA, controlling for baseline marginal adaptation scores, revealed statistically significant main effects for both groups (p < 0.001) and subgroups (p < 0.001). However, the group × subgroup interaction was not significant (p = 0.913), indicating that the effect of the group factor was consistent across different subgroups and vice versa. The covariate, baseline marginal adaptation scores, also had a significant influence on the outcome (p = 0.048). The inference is that there are substantial and independent differences in the final marginal adaptation scores attributable to the primary group classification (e.g., crown type) and secondary subgroup classification (e.g., luting cement type). The lack of a significant interaction suggests that the performance of one group does not depend on or change across the subgroups (Table 5).

**Table 5:** ANCOVA comparing marginal adaptation scores at three months between groups, adjusted for baseline scores. ANCOVA, adjusted for baseline scores; Group = crown type (metal-ceramic vs. all-ceramic), Subgroup = cement type (resin-modified glass ionomer vs. self-adhesive resin cement). Source: Scoring system for marginal adaptation was taken from a previous study by Felton et al. [10]. *Statistically significant at p < 0.05. ANCOVA: analysis of covariance

Variable	Sum of squares	df	Mean square	F-value	p-value
Group	9.6	1	9.6	18.99	0.001*
Subgroup	11.19	1	11.19	22.14	0.001*
Group x subgroup	0.01	1	0.01	0.01	0.913
Baseline marginal adaptation scores	2.06	1	2.06	4.08	0.048*

## Discussion

The findings of this clinical study highlighted notable differences in the marginal adaptation of metal-ceramic and all-ceramic FPDs over a short-term follow-up period, with the choice of luting cement playing a pivotal role in the outcomes. All-ceramic restorations demonstrated better overall marginal integrity, particularly when paired with self-adhesive resin cement, whereas metal-ceramic FPDs showed more pronounced degradation, especially with RMGIC. These results underscore the interplay between restorative material properties and cementation techniques in influencing early clinical performance.

Previous research has supported the superior marginal adaptation observed in all-ceramic systems. For instance, a comparative analysis of marginal fit in metal-ceramic and zirconia-based posterior FPDs using in vitro measurements found that zirconia frameworks often exhibit tighter marginal seals because of the precision of CAD/CAM fabrication, which minimizes the distortions common in traditional casting methods for metal-ceramic restorations [11]. This aligns with our observations, as CAD/CAM-processed zirconia in the all-ceramic group likely contributed to the enhanced initial fit and resistance to early marginal breakdown. Similarly, Gonzalo et al. [12] reported superior marginal adaptation of FPDs fabricated with zirconia systems, with no statistical difference between pre- and post-cementation. This advantage stems from zirconia's biocompatibility and lower thermal conductivity, which reduces microleakage and thermal expansion mismatches at the tooth-restoration interface, compared to metal alloys [13]. In contrast, durable metal-ceramic systems are prone to porcelain chipping or marginal discoloration over time, potentially exacerbating discrepancies, as seen in our metal-ceramic cohorts [3].

Regarding luting agents, self-adhesive resin cement outperformed RMGIC across both restoration types, which is consistent with studies evaluating cement performance in zirconia-based prostheses. A clinical comparison of RMGIC and self-adhesive resin cement for full-coverage zirconia restorations found that both provide reliable outcomes; however, self-adhesive variants offer improved sealing properties, leading to fewer marginal issues in the early postoperative phase [14]. This is attributable to the dual-cure mechanism and hydrophobic nature of self-adhesive resins, which enhance bond strength and reduce solubility in the oral environment, thereby minimizing gap formation and secondary caries risk [15]. In vitro evaluations further corroborate this, showing that self-adhesive resin cements exhibit lower microleakage than RMGIC in zirconia crown systems, owing to better chemical adhesion to both the tooth structure and ceramic surfaces [16]. The inferior performance of RMGIC in our study may be related to its higher water absorption and fluoride release, which, while beneficial for caries prevention, can lead to dimensional changes and weakened marginal seals under occlusal stress. Notably, the lack of a significant interaction between crown type and cement suggests independent effects, implying that, while all-ceramic materials inherently support better adaptation, cement choice can optimize or compromise outcomes regardless of the restoration material.

These results are also in line with the broader literature on the functional parameters of metal-ceramic versus all-ceramic restorations. A literature review assessing survival rates and complications indicated that all-ceramic FPDs have fewer technical failures related to marginal integrity, particularly in posterior regions, owing to advancements in material strength and esthetics [17]. However, some studies reported no significant differences in marginal fit between all-ceramic and conventional metal-ceramic systems, or between pressed-to-metal restorations and all-ceramic, suggesting that fabrication techniques and operator variability play crucial roles [18]. Discrepancies across studies may arise from variations in assessment methods. While our use of modified USPHS criteria focused on clinical detectability, laboratory-based studies often employ microscopic measurements, which may overestimate clinical relevance.

Clinically, these findings have important implications for prosthodontic practice. The preference for all-ceramic FPDs with self-adhesive resin cement could enhance longevity and reduce the need for early intervention, particularly in esthetically demanding or periodontally sensitive cases. This combination minimizes biological risks, such as plaque retention and inflammation, promoting better patient satisfaction and oral health. Practitioners should prioritize self-adhesive resins for zirconia-based restorations to leverage their adhesive advantages and potentially decrease the incidence of marginal discrepancies that lead to costly repairs or replacements. In resource-limited settings, where metal-ceramic options remain cost-effective, selecting appropriate cements could mitigate some limitations, although transitioning to all-ceramic systems may yield long-term benefits for high-load posterior applications.

Despite these insights, this study had several limitations. The short three-month follow-up period restricts conclusions to early marginal changes, as long-term evaluations are needed to assess durability under sustained occlusal and environmental stresses. Convenience sampling and a single-center design may introduce selection bias, limiting generalizability to diverse populations or clinical settings. Additionally, while examiners were blinded, subjective elements in USPHS scoring could influence the results despite high inter-examiner reliability. The sample size, although powered for medium effects, may not detect subtle interactions, and the exclusion of complex cases (such as bruxism) overlooks real-world challenges. Moreover, the marginal fit was assessed clinically; an AI-based assessment on bite-wing radiographs could have provided better detail. Future research should incorporate longer follow-up periods, randomized multicenter trials, and advanced imaging for objective marginal assessments to validate and expand these findings.

## Conclusions

Within the limitations of this prospective clinical study, both metal-ceramic and all-ceramic FPDs demonstrated clinically acceptable marginal adaptations during the early follow-up period. However, all-ceramic restorations exhibited more favorable marginal integrity than metal-ceramic restorations. The choice of luting cement independently influenced marginal adaptation, with self-adhesive resin cement showing superior clinical performance over RMGIC irrespective of restoration type. These findings highlight the combined importance of restorative material selection and cementation strategies in optimizing the marginal adaptation and early clinical success of FPDs.
